# Photodynamic and Antibiotic Therapy in Combination to Fight Biofilms and Resistant Surface Bacterial Infections

**DOI:** 10.3390/ijms160920417

**Published:** 2015-08-28

**Authors:** Federica Barra, Emanuela Roscetto, Amata A. Soriano, Adriana Vollaro, Ilaria Postiglione, Giovanna Maria Pierantoni, Giuseppe Palumbo, Maria Rosaria Catania

**Affiliations:** 1 Department of Molecular Medicine and Medical Biotechnology, University of Naples Federico II, Naples 80131, Italy; E-Mails: federica.barra@unina.it (F.B.); emanuelaroscetto@gmail.com (E.R.); amata.soriano@libero.it (A.A.S.); vollaroadriana@libero.it (A.V.); ilariapostiglione@gmail.com (I.P.); giovannamaria.pierantoni@unina.it (G.M.P.); mariarosaria.catania@unina.it (M.R.C.); 2 Institute of Experimental Endocrinology and Oncology (IEOS), National Research Council (CNR), Naples 80131, Italy

**Keywords:** photodynamic therapy, 5-aminolevulinic acid, Gentamicin, combination therapy

## Abstract

Although photodynamic therapy (PDT), a therapeutic approach that involves a photosensitizer, light and O_2_, has been principally considered for the treatment of specific types of cancers, other applications exist, including the treatment of infections. Unfortunately, PDT does not always guarantee full success since it exerts lethal effects only in cells that have taken up a sufficient amount of photosensitizer and have been exposed to adequate light doses, conditions that are not always achieved. Based on our previous experience on the combination PDT/chemotherapy, we have explored the possibility of fighting bacteria that commonly crowd infected surfaces by combining PDT with an antibiotic, which normally does not harm the strain at low concentrations. To this purpose, we employed 5-aminolevulinic acid (5-ALA), a pro-drug that, once absorbed by proliferating bacteria, is converted into the natural photosensitizer Protoporphyrin IX (PpIX), followed by Gentamicin. Photoactivation generates reactive oxygen species (ROS) which damage or kill the cell, while Gentamicin, even at low doses, ends the work. Our experiments, in combination, have been highly successful against biofilms produced by several Gram positive bacteria (*i.e.*, *Staphylococcus aureus*, *Staphylococcus epidermidis*, *etc.*). This original approach points to potentially new and wide applications in the therapy of infections of superficial wounds and sores.

## 1. Introduction

Microbial infections are still among the leading causes of death in the world, primarily because of the surfacing of pathogenic bacteria that have developed multidrug resistance (MDR) [1]. Thus, microbial resistance to antibiotics has become a major threat to human health [2]. On the research front, it is generally recognized that major efforts are needed to discover new antimicrobial molecules [3] or, even better, alternative therapeutic approaches. Among these treatments, photodynamic therapy (PDT) has reemerged over the last decade because it proved effective against some antibiotic-resistant pathogenic bacteria [4].

PDT requires that a non-toxic photosensitizer (PS) be selectively accumulated in host cells [5,6]; the subsequent exposure of PS-filled cells to visible light of the appropriate wavelength, excites the PS and contributes to the generation of singlet oxygen and other reactive oxygen species (ROS), which cause oxidative damage and cell death [6].

Among the photosensitizers potentially amenable to be used as an antimicrobial agent, 5-aminolevulinic acid (5-ALA) presents favorable characteristics [6,7]. 5-ALA is not a PS *per se* but, when it is taken up by target cells (including bacteria), it is metabolically changed to Protoporphyrin IX (PpIX), the real photosensitizer, of which activation may lead to the required antimicrobial effects [8]. It is important to underline that the heme biosynthetic pathway is highly conserved across organisms, from non-phototrophic prokaryotes to non-plant eukaryotes [9,10].

The nature and solubility of 5-ALA makes it particularly suitable to treat wounds, or, in general, superficial skin infections. When administered locally, 5-ALA can, not only be easily absorbed by the bacterial cells, but, after its transformation into PpIX, also becomes a powerful Trojan horse.

Microbial infections sometimes appear to be more resistant to treatment because of the formation of biofilms. Such entities are a sort of multicellular communities, usually held together by a self-produced matrix in which cells are embedded within an extracellular polymeric substance (EPS) and adhere to each other and/or to a surface. The ability to form biofilms in a variety of environments is a common defensive characteristic of several bacteria. The proximity of cells within the biofilm creates the opportunity for coordinated behaviors through cell-to-cell communication using a spectrum of diffusible signals, the most well documented being the so-called “quorum sensing” [11].

Although PDT has been investigated for its ability to eradicate biofilms produced by bacteria infecting medical devices [12,13], its use, especially in combination with other approaches, has not been fully investigated as of yet.

There are several antibiotic-resistant and biofilm-producing bacteria; our study proposes an innovative treatment to eradicate infections caused by such bacteria. The treatment combines PDT with antibiotic therapy in order to achieve an additive or synergistic therapeutic effect.

## 2. Results and Discussion

The problem of bacterial antibiotic resistance is exacerbated by the strong tendency of pathogenic bacteria to form biofilms. A biofilm is an ordered conglomerate of bacteria, surrounded by a self-produced matrix composed of polysaccharides and other biological macromolecules. Chemical–physical conditions created by the biofilm make the bacteria up to 1000 times more resistant to antimicrobials than the planktonic cells; thus, bacterial biofilms sustain chronic infections, especially because they show augmented tolerance to antibiotics. The antibiotic recalcitrance of biofilm is multifactorial, and includes reduced diffusion of antibiotics, increased level of mutations, enhanced horizontal transfer of resistance determinants, and stress-activated responses inducing antibiotic tolerance [14,15]. Therefore, the need for alternative antimicrobial strategies is pressing.

Among these alternative approaches, PDT has been proposed as an antimicrobial technique to fight bacteria, including some multidrug resistant strains [6,16,17]. It is worth noting that the findings reported in a recent account [18], are that sub-lethal doses of PDT, at variance with antibiotics, do not generate resistance.

The effects and mechanisms of action of PDT in combination with antibiotics have been reported [19]. For instance, Cahan *et al.* [20] have shown that the exposure to light of Gram-negative and Gram-positive bacteria, treated with photosensitizer-antibiotic conjugates, results in good bactericidal activity. Similarly, Almeida *et al.* [21] demonstrated that PDT in the presence of antibiotics was effective in inactivating multidrug-resistant bacteria in hospital wastewaters. A few examples of the use of PDT in combination with antibiotics to fight bacterial biofilms are also found in more recent literature [22,23].

The present investigation follows along the same lines, but does not use exogenous photosensitizers, rather, an endogenously generated one is used and makes use of an ineffectual antibiotic (Gentamicin) at sub-inhibitory concentrations, which is proposed to fight bacteria organized as biofilms.

In this regard, the approach that we present in this work demonstrates that an earlier treatment of a biofilm formed by certain Gram positive bacteria with 5-ALA/PDT makes them more sensitive to Gentamicin. The reason for this may reside in the ROS-mediated destruction of—or at least significant damage to—the shield (biofilm) that prevents penetration and subsequent action of the antibiotic. This approach may have more general implications and be applicable using different antibiotics, made useless by misuse.

### 2.1. Gentamicin MIC (Minimal Inhibitory Concentration)

We assessed the minimal inhibitory concentration (MIC90) of Gentamicin that will inhibit the growth of 90% of *S. aureus*, *S. epidermidis*, and *S. haemolyticus* by micro-dilution broth assay (see Table 1).

**Table 1:** Bacterial strains’ minimal inhibitory concentration (MIC90).

Strain	MIC90 (μg/mL)
*S. aureus*	>128
*S. epidermidis*	24
*S. haemolyticus*	6

### 2.2. Biofilm Formation (Quantitative and Qualitative Aspects)

**Figure 1:**
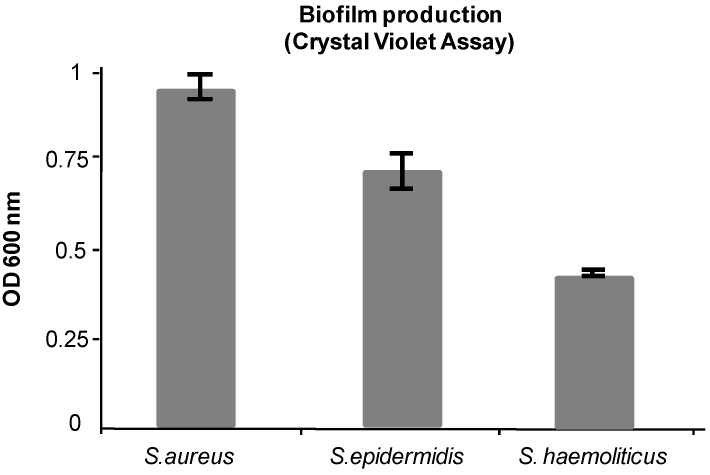
Biofilm production as determined by Crystal Violet staining. Other details in the text.

Biofilm formation was assessed by Crystal Violet staining assay [24], as described in Section 3.3. The optical densities of solubilized Crystal Violet, measured at 600 nm, were 0.95 ± 0.03, 0.72 ± 0.05, and 0.43 ± 0.005 for *S. aureus*, *S. epidermidis*, and *S. haemolyticus*, respectively (Figure 1).

Biofilms were also seen directly by confocal microscopy. In this case, bacteria were grown on coverslips. Biofilms formed by the three bacteria strains used in this study were the controls (see forward in the text).

### 2.3. Individual and Combined Therapy

#### 2.3.1. Gentamicin Treatment

Gentamicin, as standalone therapy is not suitable against the three bacteria strains as indicated by the very high MIC90.

The concentration of Gentamicin that we decided to use in combination with PDT was set at 2 μg/mL, *i.e.*, well below the measured MICs.

#### 2.3.2. Photodynamic Therapy

**Figure 2:**
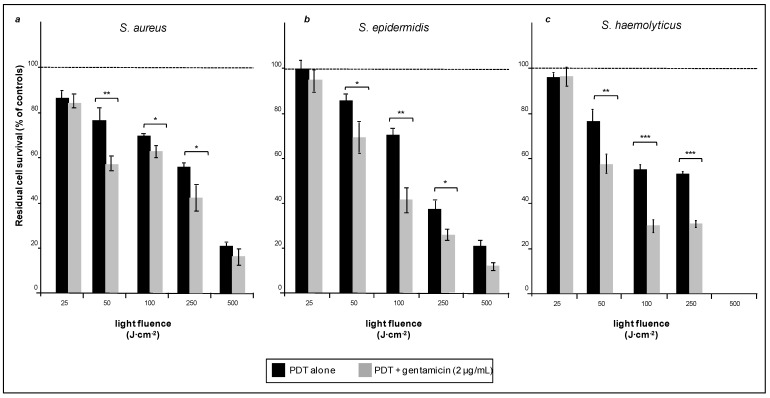
Biofilms of *S. aureus* (**a**), *S. epidermidis* (**b**) and *S. haemolyticus* (**c**) all incubated with 5-ALA and exposed to red light only (black bars) or light and Gentamicin in combination (grey bars). The dotted bar is referred to each respectively control (100%). Note (see also next figures) that the number of cells populating the biofilms is rather different within the three strains. More details in the text. * *p* < 0.05; ** *p* < 0.01; *** *p* < 0.001; NS, not significant. The bars relative to other controls (cells irradiated in the absence of 5-ALA or cell exposed to Gentamicin only) are not included in this figure.

The effects of photodynamic therapy on bacterial viability within biofilms were evaluated after preincubation with 5-ALA and irradiation with light fluences, from 25 to 500 J·cm^−2^. Despite the shielding of biofilm, the viability of all types of cells analyzed decreased with the increase of the light dose administered. Two of the three bacterial strains used, namely *S. aureus* and *S. epidermidis*, were rather resistant to treatment, thus, a fluence of about 500 J·cm^−2^ was necessary to abate their viability to 20% of the original value (Figure 2a,b, black bars). The third strain *S. haemolyticus*, was apparently more sensitive to low doses of PDT since a fluence as low as about 100 J·cm^−2^ was sufficient to abate bacterial viability by a factor close to 2. However, viability of this strain did not abate any further, even at a fluence as high as 250 J·cm^−2^ (Figure 2c, black bars).

In all experiments, the temperature of medium during exposure to light was kept below 28 °C as indicated in the Experimental Section.

#### 2.3.3. Combined Treatments

The effects of the addition of Gentamicin (2 μg/mL) on biofilms and bacterial viability after irradiation from 25 to 500 J·cm^−2^ were evaluated quantitatively by XTT (2,3-bis-(2-methoxy-4-nitro-5-sulfophenyl)-2H-tetrazolium-5-carboxanilide) assay and qualitatively/semi-quantitatively by confocal laser scanner microscopy (CLSM).

##### Quantitative Aspects

The XTT assay demonstrated that photodynamic treatment (500 J·cm^−2^) of the strongly resistant *S. aureus* reduced its viability to about 20%, the latter was reduced even further when bacteria pre-exposed to PDT were treated with Gentamicin (2 μg/mL). The drops in cell viability brought about by the combined treatment (Figure 2a, gray bars) were not dramatic, but measurable and highly reproducible. More interesting results were obtained with *S. epidermidis*, of which exposure to a fluence of 500 J·cm^−2^, followed by Gentamicin (2 μg/mL) reduced the bacterial viability to about 10% (Figure 2b, gray bars). The biofilm formed by *S. haemolyticus* was particularly sensitive to combined treatment, as Gentamicin (2 μg/mL) applied to a biofilm exposed to a fluence as low as 100 J·cm^−2^ (Figure 2c, gray bars) reduced bacterial viability to close to 30%. For each bacterial strain, we have also measured the viability after exposure to light (without 5-ALA) and exposure to Gentamicin alone. The measured values were essentially undistinguishable from those of untreated cells, and were not reported in Figure 2.

##### Qualitative/Semiquantitative Aspects: Confocal Laser Scanner Microscopy (CLSM) Micrographs and Biomasses

An indisputable demonstration of the increase in the effectiveness of the antimicrobial action of PDT when in combination with Gentamicin, is offered by the visual inspection of the biofilms (before and after individual or combined treatments) that can be made using CLSM.

**Figure 3:**
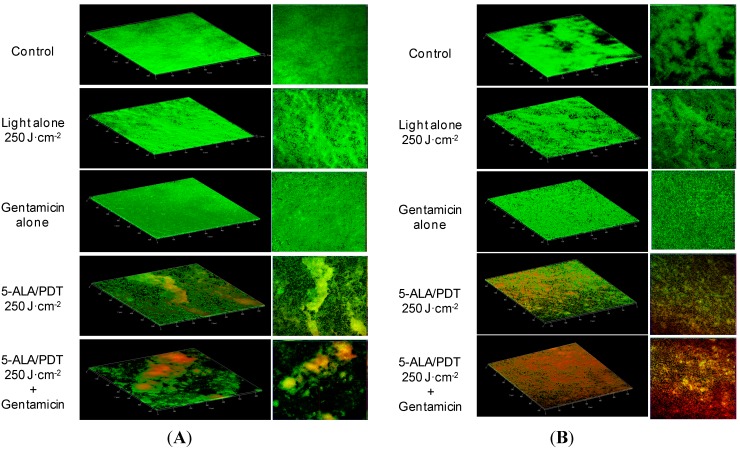

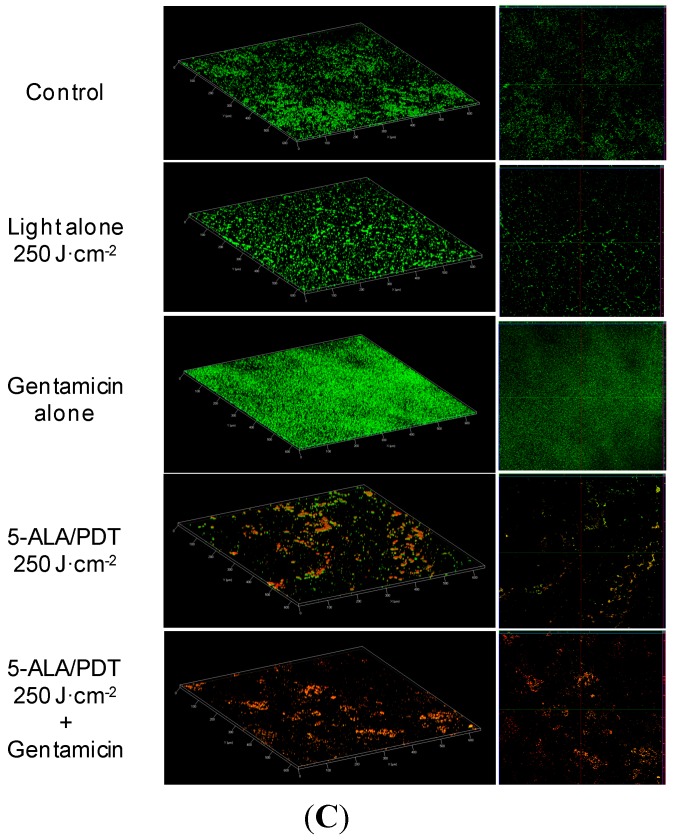
(**A**) Confocal laser scanner microscopy (CLSM) micrographs: three dimensional (**left panels**) and orthogonal reconstructions (**right panels**) of the biofilm formed by *S. aureus*. The pictures refer to the various experimental conditions as indicated on the left. Light fluence was set at 250 J·cm^−2^, Gentamicin concentration at 2 μg/mL. The fluorescence is associated with live (green) and dead (red) cells, respectively; (**B**) CLSM micrographs: three dimensional (**left panel**) and orthogonal reconstructions (**right panel**) of the biofilm formed by *S. epidermidis*. The pictures refer to the various experimental conditions as indicated on the left. Light fluence was set at 250 J·cm^−2^, Gentamicin concentration at 2 μg/mL. The fluorescence is associated with live (green) and dead (red) cells, respectively; (**C**) CLSM micrographs: three dimensional (**left panels**) and orthogonal reconstructions (**right panels**) of biofilm formed by *S. haemolyticus*. The pictures refer to the various experimental conditions as indicated on the left. Light fluence was set at 250 J·cm^−2^, Gentamicin concentration at 2 μg/mL. The fluorescence is associated with live (green) and dead (red) cells, respectively. Scale bars represent 100 μm as indicated in micrographs.

Figure 3A–C depict micrographs of biofilm formed by *S. aureus* (A), *S. epidermidis* (B), and *S. haemolyticus* (C), the effects caused by mono and combined therapy (5-ALA/PDT and 5-ALA/PDT followed by Gentamicin) and other controls. In particular:

Figure 3A depicts a very compact biofilm formed by *S. aureus*, which covers virtually all the cover-glass. While exposure to light alone or Gentamicin alone did not cause any major change in the biofilm, 5-ALA/PDT caused some disruption, as shown by the differential staining with SYTO9 (green fluorescence, live cells) and propidium iodide (red fluorescence, dead cells). If PDT alone was able to reduce the viability of *S. aureus* cells, PDT followed by Gentamicin (2 μg/mL) increased biofilm detachment and decreased cell viability.

The biofilm formed by *S. epidermidis* was rather dense covering the major part of the surface of the cover-glass. Even in this case, exposure to light alone or Gentamicin alone did not cause any significant change in the biofilm structure and cells viability. In contrast, 5-ALA/PDT caused partial detachment of the biofilm and bacterial death (red fluorescence), especially within the biofilm outer layers (the reduced size of the figures do not allow to appreciate in full this detail). The combined treatment enhanced the harmful effects (Figure 3B).

Compared to the other *Staphylococcus* strains, *S. haemolyticus* formed a differently structured biofilm, in that it consisted of individual clusters widely dispersed on the cover-glass surface. Indeed, exposure of biofilm to the red light (without 5-ALA) did not cause any change in the biofilm and cell viability. Interestingly enough, the exposure of this biofilm to Gentamicin alone induced an apparent increase in the biofilm density. In contrast, 5-ALA/PDT induced detachment of micro-colonies while viability of outer layer cells was strongly reduced. Finally, the exposure of the *S. haemolyticus* biofilm to a combined treatment resulted in the elimination of most colonies since only very few clusters of bacteria could be seen. In accord, the number of surviving bacteria (green) was strongly reduced (Figure 3C).

Biomasses were roughly evaluated by means of the IMARIS v7.0 software (BITPLANE, Belfast, UK). This software measures the volumes of biofilms (μM^3^) relative to a reference surface. Comparing these results with those obtained by the Crystal Violet assay, we found a satisfactory agreement.

## 3. Experimental Section

### 3.1. Bacterial Strains and Culture Conditions

Three clinical isolates of the *Staphylococcus* genus were included in the study, namely *S. aureus*, *S. epidermidis*, and *S. haemolyticus*. Bacteria were obtained from collection of the Laboratory of Clinical Microbiology, University Federico II of Naples, and were identified by biochemical characteristics using the VITEK II system (bioMerieux, Marcy l’Etoile, France), and confirmed by MALDI TOF MS (Bruker Daltonics, Macerata, Italy). Bacterial strains were selected on the basis of Gentamicin resistance (MIC > 1 μg/mL), determined by automatic antimicrobial susceptibility testing system (VITEK II).

Bacteria were grown at 37 °C for 24 h in Brain-Hearth Infusion (BHI; Becton Dickinson, Buccinasco, Milan, Italy) broth, and aliquots were frozen in BHI-glycerol at −80 °C until use.

### 3.2. Determination of MIC (Minimal Inhibitory Concentration) of Gentamicin

MIC value of Gentamicin for each strain was determined by micro-dilution test, according to the European Committee on Antimicrobial Susceptibility Testing (EUCAST version 4.0, 1 January 2014).

*Staphylococcus* cultures were grown in BHI broth in test tubes overnight at 37 °C in a rotary shaker (200 rpm). The cultures were then diluted and adjusted to the final concentration of 5 × 10^4^ CFU/well. The multiwell plates were incubated at 37 °C for 24 h. Microorganism growth was determined by reading the absorbance at 630 nm in a plate reader, and the minimum inhibitory concentration was considered as the lowest concentration at which at least 80% of growth was inhibited. All tests were performed in triplicate. The concentrations of Gentamicin tested spanned from 0.25 to 128 μg/mL.

### 3.3. Evaluation of Biofilms in Vitro

Biofilm production by *Staphylococcus* strains was assessed using an adherence assay on 96-well tissue culture plates, as described previously by Stepanović *et al.* [25].

Briefly, a single colony from overnight cultures of bacterial strains was adequately diluted in trypticase soy broth (TSB) to adjust the turbidity of the bacterial suspension to 0.5 McFarland (corresponding to approximately 10^8^ CFU/mL). This suspension was further diluted 1:100 in TSB with 10% (*w*/*v*) glucose and transferred in a multiwell plate (200 μL/well). Negative controls were constituted by broth only. The multiwells were then incubated in aerobic conditions. After 24 h at 37 °C, the content of each well was aspirated and the wells gently washed twice with sterile phosphate buffered saline (PBS) to remove exclusively non-adherent cells. The adherent biofilms were fixed (dried) incubating the plates at 60 °C for 1 h.

Biofilms were stained with 1% Crystal Violet (30 μL/ well) for 15 min and then washed with 150 μL of sterile PBS. Finally the dye was solubilized with 150 μL of 95% ethanol per well for 20 min. The optical density of solution in each well was measured at 600 nm using a microplate reader (Model 680, Bio-Rad, Segrate, Milan, Italy). Based on the optical densities of bacterial biofilms, all strains were all classified according to Stepanović [17] as strong biofilm producers.

### 3.4. Photosensitizer and Light Source

5-Aminolevulinic acid (5-ALA) was obtained from Sigma Life Science. A stock solution of 5-ALA (300 mM) was prepared in sterile water and the solution was stored at −20 °C in the dark.

Before each experiment, 5-ALA was appropriately diluted in sterile saline solution to obtain the fixed desired concentration of 40 mM [26] in sterile BHI broth and incubated in aerobic conditions at 37 °C for 24 h. For 5-ALA activation, bacteria were irradiated using a Light-Emmitting Diode (LED) array (S-630, Alpha Strumenti, Melzo, Italy) designed specifically for photodynamic therapy. The apparatus emits a red narrow-band light and is constituted by a rectangular array of 50 LEDs. The maximum emission peak is centered at 630 nm. Full width at half maximum (FWHM) spectral bandwidth of the red LED is less than 30 nm.

The light source was placed at a distance from the plates (10 cm) to ensure uniform illumination of the entire biofilm. Fluence rate at level of the plate was fixed at 5 J·cm^−2^/min and light doses were irradiated to 500 J·cm^−2^.

### 3.5. Individual Treatment

The formation of biofilms was obtained by incubating bacteria in aerobic conditions for 24 h in 96-wells microplates. After the incubation, wells were washed with PBS and the culture medium was substituted with 100 μL of 5-ALA 40mM in sterile BHI broth and incubated overnight. After incubation, the liquid phase containing the prodrug was discarded and replaced with 100 μL of PBS. The procedure of irradiation consisted in exposing the plates to light fluences ranging from 25 to 500 J·cm^−2^. Each plate contained multi-samples of bacterial strains (*i.e.*, *S. aureus*, *S. epidermidis*, and *S. haemolyticus*), so that each bacterial strain, exposed to a single light fluence, received exactly the same light dose. To avoid possible uncontrolled increase of the temperature during long irradiation (especially at higher fluences), microplates were partially immersed in bath with flowing tap water. Temperature was checked regularly.

### 3.6. Combined Treatment

For combined treatment, after irradiation plates were washed with PBS and incubated with 200 μL/well of Gentamicin sulfate salt stock solution (2 μg/mL) (Sigma Life Science, Milano, Italy) overnight at 37 °C.

For each bacterial strain, three different controls were included: untreated cells, cells exposed to light (without 5-ALA), and cells exposed to Gentamicin (2 μg/mL) alone.

### 3.7. XTT (2,3-Bis-(2-methoxy-4-nitro-5-sulfophenyl)-2H-tetrazolium-5-carboxanilide) Assay

After individual or combined treatments, wells were washed with PBS and the residual viabilities associated to biofilms were evaluated by means of a classical XTT reduction assay [27]. The XTT cell proliferation kit II was obtained from Roche Diagnostics (Milan, Italy).

In our case, the XTT salt was dissolved in sterile BHI broth and incubated in the dark at 37 °C for 3 h.

The assay is based on the cleavage of the yellow tetrazolium salt XTT to form an orange formazan dye, which may occur exclusively by means of viable cells. The increase in the formazan dye production (measurable spectrophorometrically at 490 nm) is proportional to the effective number of live biomass. Optical densities at 490 nm were collected with an ELISA plate reader (Bio-Rad, Segrate, Milan, Italy). Data (from at least triplicate samples) are expressed as percentage of treated cells *versus* untreated controls.

### 3.8. Confocal Laser Scanning Microscopy (CLSM) Assay

Biofilm production was monitored by letting cells grow on Nunc^®^ Lab-Tek^®^ II chambered cover-glasses (Sigma Life Science, Milano, Italy). Biofilms were stained with LIVE/DEAD BacLight Bacteria Viability stains (Life Technologies, Monza, Italy) according to manufacturer instructions. This kit consists in two nucleic acid stains: SYTO 9 and propidium iodide (PI). The first, SYTO 9, penetrates both viable and nonviable bacteria. In contrast, PI penetrates bacteria with damaged membranes (*i.e.*, nonviable cells) while quenching fluorescence emitted by SYTO 9. Dead cells, emit the PI red fluorescence; viable cells, emit green light. Images were captured using LSM 710 inverted confocal laser-scanning microscope (Zeiss, Arese, Milano, Italy), while quantitation of residual biomasses was performed using the IMARIS v7.0 software package (from BITPLANE, Belfast, UK).

### 3.9. Statistical Analysis

All experiments were performed in triplicate and repeated three times. Significance was assessed by Student’s *t*-test for unpaired independent data for comparisons between two means (one-tailed), using the free software “R Development Core Team” [28]. *p* values: * *p* < 0.05; ** *p* < 0.01; *** *p* < 0.001.

## 4. Conclusions

Skin and soft tissue lesions resulting from burns, trauma, surgery, or coagulopathies, can easily become infected especially by opportunistic bacteria. Many of these infections are hospital-acquired and caused by multi-drug resistant pathogens. Biofilm growth phenotype has been implicated in many superficial infection complications, favoring the microbial persistence and delaying the lesion resolution.

PDT has been proposed as alternative antimicrobial therapy for the treatment of localized bacterial infections. Several authors have investigated the ability of PDT to inactivate bacteria growing as biofilm, but most of the studies regard oral cavity bacteria. As already underlined in the introductory part of this paper, there are numerous reports that propose PDT as a potential general tool to fight bacteria, either as standalone therapy or in combination with antibiotics. However, they all use antibiotics at high concentrations and often study the effects of the combination on planktonic bacteria. The present work exploits the properties of a naturally occurring metabolite (5-ALA), which generates the active photosensitizer inside the bacterial cells, uses Gentamicin at low concentration, and reports qualitative and quantitative effects on bacterial cells organized as biofilms.

Gentamicin is indicated in the treatment of skin and soft tissue infections by Gram-negative bacteria and Staphylococcus species. As most aminoglycosidic antibiotics, Gentamicin grounds its activity on its capacity to tightly bind the 30S subunit of the bacterial ribosome. As a consequence of this interaction, protein synthesis is impaired and bacteria cells die. The fundamental “pre-condition” for Gentamicin to exert its activity (similarly to all other aminoglycosides) is the possibility of penetrating the membrane of bacteria. This becomes more difficult when bacteria have formed a biofilm. The resistance, however, is not only attributable to a reduced Gentamicin uptake or decreased cell permeability, since it is known that some resistant bacteria may make inoffensive the antibiotic taking advantage by specific aminoglycoside modifying enzymes. Another, indeed unusual mechanism, is the possible alteration of the antibiotic ribosomal binding sites.

PDT works by forming large amounts of ROS inside the cells. The bursts of ROS production determined by the exposure of endogenously produced PpIX to light generate local and profound damage. ROS make protein breakdowns and cross-linking, modify sugars, peroxidate lipids profoundly damaging membranes, harming ribonucleic acids (both sugars and bases). Our images by confocal microscopy obtained through direct observation of biofilms (formed by all three strains studied) after 5-ALA/PDT, demonstrate that the bacteria intake and metabolize 5-ALA and that the neo-synthesized photosensitizer (PpIX) rapidly spreads within the cells. The data on cell viability and the confocal microscopy images that we have collected after exposure of biofilms to light, undoubtedly document the 5-ALA/PDT effectiveness. However, this is not all. Although ROS production is responsible of the death of the major part of bacterial cells, it is conceivable that a number of cells is only damaged and would survive. Most of these cells, however, are probably not void of detrimental effects: they would present certainly some ROS-mediated membrane ruptures and/or non-specific impairment of endogenous enzymatic activities. Given the biosynthetic nature of the matrix forming the biofilm, this may be severely impaired. In our view, a model shapes up in which Gentamicin added to bacterial biofilms after photodynamic treatment, penetrates bacteria more easily and/or is not degraded, prior to exerting its action, because most intracellular enzymes may have been damaged by oxidation.

It is clear that we have explored only a limited number of resistant (Gram positive) strains and the situation cannot be generalized as the effectiveness of 5-ALA/PDT depends on the capacity of 5-ALA to enter the cell and to be transformed in PpIX. Both processes differ in different bacteria, strongly conditioning the treatment outcome. In addition, we do not know about the presence of potential transporters capable of extruding neo-synthesized PpIX. However, the improvement in therapeutic efficacy against the biofilm that we have observed nicely fits with the model that we have proposed. This hypothesis, however, deserves further studies.
